# COVID-19 exit strategy: Transitioning towards a new normal

**DOI:** 10.1016/j.amsu.2020.09.046

**Published:** 2020-10-02

**Authors:** Shahrizan Jamaludin, Nor Azali Azmir, Ahmad Faisal Mohamad Ayob, Nasharuddin Zainal

**Affiliations:** a Department of Maritime Technology, Faculty of Ocean Engineering Technology and Informatics, Universiti Malaysia Terengganu, Malaysia; b Department of Engineering Mechanic, Faculty of Mechanical and Manufacturing Engineering, Universiti Tun Hussein Onn, Malaysia; c Department of Electrical, Electronic and Systems Engineering, Faculty of Engineering and Built Environment, Universiti Kebangsaan Malaysia, Malaysia; d Renewable Energy and Power Research Interest Group (REPRIG), Eastern Corridor Renewable Energy Special Interest Group, Faculty of Ocean Engineering Technology and Informatics, Universiti Malaysia Terengganu, Malaysia; e Marine Materials Research Interest Group, Universiti Malaysia Terengganu, Malaysia; f Naval Architecture Research Interest Group, Faculty of Ocean Engineering Technology and Informatics, Universiti Malaysia Terengganu, Malaysia

**Keywords:** COVID-19, SARS-CoV-2, New normal, Safety and health, Soft landing approach

## Abstract

The COVID-19 outbreak from the SARS-CoV-2 virus has shocking us with its fast transmission and deadly complication. Due to that, the movement restriction has been enforced to contain this pandemic. Recently, there is an increasing pressure to restart and resurrect social and economic sectors, and to allow people to get back to work. This must be well planned before the movement restriction is lifted. Because of that, this paper aims to review and make recommendations on the new normal for our daily activities and works. Firstly, the social and economic sectors must be opening in phases by emphasizing safety and health than an economic recovery. In the meantime, the WHO recommendations on social distancing and personal hygiene must be adapted and become a new normal. Because of that, the government and local authorities should develop a soft landing approach based on the WHO recommendations. Next, the community must be engaged and empowered to do their parts in preventing the spread of COVID-19. From the new normal recommendations, the people can continue their daily routines, and at the same time can reduce COVID-19 transmission. However, medical possibilities are not considered while compiling these new normals and procedures. The population must adapt and embrace the new normal to control, reduce and prevent the spreading of COVID-19, as it could be with us for a long time.

## Highlights

• The new normal and procedures for our daily activities and works during COVID-19 pandemic.
• The new normal in workplace, industry, health care service and elderly care service.
• The community must embrace new normal for their own safety and health.

## 1 Introduction

The coronavirus disease 2019 (COVID-19) is transmitted from the release of droplets when an infected person coughs or exhales. Then, the released droplets will fall on the nearby objects and surfaces, thus contaminating them. A nearby person can be infected by touching fomites, and then touching their mouth and nose [1]. Moreover, the virus itself has the potential for viral entry from the human eyes [2]. The common symptoms of COVID-19 are mild fever, dry cough, shortness of breath and muscle pain [3]. However, severe symptoms such as high fever, pneumonia and acute kidney failure can be observed in the older persons and persons with comorbidities [4]. Bear in mind that anyone could be in contact with COVID-19. Furthermore, this virus can cause severe complications to a high-risk group such as children, older persons, persons with comorbidities, persons with a weak immune system, pregnant women, and disabled persons [5]. Because of that, vulnerable people must be protected at all costs as the disease can be deadly to them. In addition, most of the COVID-19 patients show no symptoms [6,7]. COVID-19 patient with no symptoms is known as silent spreader [8,9] and can spread the disease unknowingly through physical contact and close conversation.

There are three ways to stop the spreading of COVID-19 [10]. One involves the movement restriction on social and economic sectors. Secondly, an efficient vaccine is developed, which may take at least 12–18 months for clinical trials and mass production [11]. The third way is to stop the COVID-19 outbreak naturally with herd immunity [10].

Countries such as Taiwan, South Korea and Sweden are not implementing movement restriction, as they are relying on a high level of self-regulation and self-discipline among their citizens. The citizens have been engaged and empowered to do their parts in preventing the spread of COVID-19. Meanwhile, Malaysia and Singapore implement both movement restriction and self-regulation approaches. In Malaysia, movement restriction is implemented in the first few weeks to contain the outbreak. Once the COVID-19 infection curve has been flattened, the self-regulation and self-discipline approaches are implemented to restart and recover the social and economic sectors.

Movement restriction puts a lot of pressure on the population's physical, mental, health, social and economy. Moreover, global economies are hardly hit by the COVID-19 outbreak [12]. Hence, there is an increasing pressure to restart and resurrect the social and economic sectors, and to allow people to get back to work. This must be well planned before the movement restriction is lifted. The developed procedures should find the right balance between economic resurrection and COVID-19 prevention [13]. The social and economic sectors must open in phases by emphasizing safety and health than an economic recovery.

Hence, this paper reviews and recommends new normals and procedures for daily activities and work-lives to protect people's safety and health. Medical possibilities are not considered while compiling these new normals and procedures. This soft-landing approach is crucial to restart and resurrect the social and economic sectors. No ethical approval required in this study.

## 2 Responsibility

The World Health Organization (WHO) has recommended six criteria for countries that consider lifting the movement restriction. The first criterion is the COVID-19 transmission in a country has been controlled. The second criterion is the health care facilities including hospitals and clinics are able to find, test, isolate, treat and trace every close contact. The third criterion is the transmission risk for a high-risk group has been minimised such as in the health care facilities and nursing homes. The fourth criterion is the preventive measures are in place in the essential places such as kindergartens, schools, shops and workplaces. The fifth criterion is the imported COVID-19 transmission risks such as from the travellers, tourists and foreign students can be managed. The final criterion is the communities have been prepared for a new normal [14].

The WHO recommends social distancing and personal hygiene to be adopted by the population. Social distancing or physical distancing is a safe distance between individuals to prevent respiratory droplet transmission while working and social interactions [15]. Meanwhile, personal hygiene is a good hand-washing practice as in Ref. [16]. Moreover, a facemask is recommended to be used if the mass gatherings and crowded places are unavoidable [17,18]. A study reported that the cotton or cloth mask could prevent the spread of viral load up to 96% [19]. Furthermore, it shows a strong signal to the community that the current pandemic is still ongoing [20].

The government must develop the standard operating procedures (SOPs) for every industry based on the WHO recommendations. The economic sectors open in phases, by prioritising the most essential businesses. Besides, the government should consider developing contact tracing and geofencing systems. Contact tracing is a system to trace people who have physical contact and close conversation for a prolonged period with an infected person. Geofencing refers to the use of application to establish a virtual fence that limits the movement of an infected person, hence alarming the authority if the virtual fence is disregarded. This system requires active monitoring using location-based applications tied with the bearer. Moreover, the close contacts of the suspected person can be traced, thus isolation and diagnostic testing can be done on them. Close contact is being closer than 1 m apart for more than 15 min with an infected person [18]. Furthermore, the government should consider enforcing a strict law for those who are violating COVID-19 preventive measures [21].

The procedures introduced by the government must be enforced by the local authorities to ensure the safety and health of the people. The authorities can also prepare a list of dos and don'ts to help the people from contacting this coronavirus. Moreover, the local authorities should disinfect high-risk areas such as public parks and public transports. COVID-19 cleaning and disinfecting practices are referred to Ref. [22].

Occupational safety and health (OSH) professionals can help and advise their employers regarding the COVID-19 issues to maintain the right balance between health and business operations. The OSH professionals are not only protecting the workers but also the clients, visitors and customers. They can also plan and enforce the COVID-19 safety measures in their workplaces.

Meanwhile, the employers must ascertain the essential and non-essential works in their operations. After that, an adequate number of workers for essential operations can be organised. Employers who are unable to comply are advised to remain close and work from home. Moreover, the employers should adapt and embrace the industry 4.0 technologies such as artificial intelligence, internet of things, big data, virtual reality, halography, cloud computing, autonomous robot, 3D scanning, 3D printing and biosensor which can reduce contacts among their employees [23].

Other than that, people must be responsible for themselves and their families. A person should go out or travel only when it is necessary such as to get medical treatment, groceries, foods, necessities, essential works and emergency situations [24], and must return home promptly. It is well recommended that only one person is allowed to go out at a time. The person should disinfect his or her shoes or leave them outside before entering their houses, as the coronavirus could be present on the floor surfaces. Then, a person should go straight to the bathroom to take a bath with water and soap, before touching their family members. Clothes must be changed and washed upon returning home.

## 3 New normals and procedures

Rapid diagnostic, drug and vaccine are not the only COVID-19 exit strategy. Self-regulation and self-discipline are equally important to prevent the virus from reappearing in the future [25]. The people must regulate themselves by following procedures provided by the government and authorities. Besides, people must discipline themselves to avoid violating the procedures. Temperature checking will become a new normal or routine as an initial measure for COVID-19 detection [26,27]. The persons with 37.5 °C or above temperature will be denied entry, and must seek medical attention immediately.

The other normal is the organisers, workers, visitors and participants at the meetings, events and gatherings must provide their contact details such as their name, telephone number, email and home address. These pieces of information will be shared with the health authorities if COVID-19 infection happens in the respective places.

However, if COVID-19 infection happens, the respective workplaces, premises and buildings must be shut down immediately and all operations are suspended. The health authorities must be informed and contact details will be shared for contact tracing. Then, the diagnostic test is implemented on all workers and visitors. A person must self-isolate, be treated, and not be allowed to come to work after being infected. The infected person can come to work after at least 11 days of self-isolation. This is because the infected person is no longer infectious after 11 days of getting sick [28]. The workplaces can be opened after being disinfected.

### 3.1 Working from home

Working from home has become a new option for employers to operate during the outbreak. Some employers are encouraging their workers to work from home as long as they are comfortable and necessary [29]. Moreover, working from home can prevent workers from contacting the COVID-19 infection outside of their homes. The workers with comorbidities are advised to work from home as much as possible. However, it is equally important to protect the workers’ physical and mental healths by providing them the appropriate tools and environment to work from home.

A worker must stick to the same working time for example from 8 a.m. to 5 p.m. During working time, a worker must take intermittent breaks to stretch and do light exercises for 15 min, every 2 h. Breathing in fresh air has many advantages such as improving heart rate and blood pressure, giving sharper mind, cleaning lungs and providing energy.

A dedicated workspace must be prepared for working from home. The workspace must be comfortable, quiet, has mild temperature and good lighting. Moreover, it must be an uninterrupted space and separated from the rest of the house. This is important to avoid distractions from their family during the working hours. The workspace could be in a garage, basement, veranda or bedroom, as long as it is comfortable.

Nevertheless, full productivity is not expected and realistic since each worker needs to adapt to a new normal. Some workers might experience anxiety and stress when starting to work from home. However, productivity can be improved over time. The given tasks and assignments can be completed efficiently. Moreover, the geofencing can be used to enable and inform employers about the whereabouts of their workers. This is to ensure the workers commitment towards their tasks.

Recently, there is increasing evidence that domestic animals such as cats can spread the virus to the other cats [[30], [31], [32]] and show no symptoms [33]. Previously, the SARS-CoV-2 virus is originated from bats and transmitted from animals to humans [34]. Therefore, precautions must be taken to prevent transmission from pets to humans. Pets must be quarantined to prevent physical interactions with other animals outside.

### 3.2 Workforce and workplace

It is recommended for the employers to resume operations with half of their workforce. This can prevent a large presence of workers in the workplace, thus can reduce physical contact among them. Only essential works are done in the workplace, while others should work from home. Flexible working and business hours are recommended to avoid a large crowd in public transports during peak hours. Moreover, the workers can work on alternate days in the office. Furthermore, a four-day workweek has been encouraged in New Zealand and Japan to boost the struggling economy and increase productivity [35]. Therefore, this will allow people to travel during their off days and boost the local economy.

The employers must promote and enforce social distancing, personal hygiene and personal protective equipment (PPE) among their workers. Everybody must adhere to social distancing at least 1 m apart. This is to ensure no physical contacts and close conversations. Moreover, the hand-washing facilities can be accessed by workers, contractors and visitors. Hand sanitisers must be provided at all entrance points. On the other hand, a good cough etiquette is emphasised if a face mask is unavailable. Besides, the geofencing system can be used to ensure social distancing is observed by the workers. The workers who are violating social distancing will be warned by their employers.

The face-to-face meeting can be substituted with an online meeting. However, the face-to-face meeting should be short, if it is unavoidable. Moreover, the workers are encouraged to telework between departments, regional offices and international offices. Work travel should be limited to essential works only. On the other hand, handshaking is not encouraged to avoid close contact.

The workers must clean and disinfect their workplace to prevent the spreading of COVID-19. The virus itself can be transmitted through fomites such as handle, doorknob, tabletop, lift button, lamp switch, fan switch and rail. The virus can remain infectious on these surfaces up to 9 days [36]. However, these fomites can be insulated with copper as the virus is not viable on its surface [37]. The floor should be disinfected too as there were cases where the virus is detected on the shoes of the health care workers [38]. Furthermore, toilet equipment such as tap, sink, door, hand rail, toilet seat and toilet cover must be disinfected regularly. Preventive maintenance on the ventilation system must be carried out periodically. The ventilator filter must be replaced to prevent cross contamination from bacteria and viruses. In fact, the previous coronavirus outbreak (SARS) was transmitted through an airborne transmission from the toilet flush and an exhaust fan of the plumbing system [39]. Because of that, efficient air circulation and ventilation must be maintained to ensure people's safety. Also, it is recommended to avoid crowded places such as the canteen and cafe during the lunch hour. The workers can order their meals for takeaway and enjoy their lunch at their desks.

On the other hand, workers with COVID-19 symptoms must stay at home and notify their employers. The sick worker must avoid close contact with others and seek medical attention immediately. Furthermore, an emergency plan must be carried out when the worker shows COVID-19 symptoms during the working hours. Prompt action to be taken by isolating in a dedicated room. This dedicated room must have good ventilation and a necessity for the suspected worker. The suspected worker is not allowed to leave the room. Then, the employer must contact the health authority immediately. Specific procedures must be taken to transfer the suspected worker from the workplace to the hospital. If the worker is infected, the whole household members must self-isolate. Then, the health authority will conduct a diagnostic test for household members. The household members are not allowed to leave the house until the result is obtained.

### 3.3 Manufacturing, construction and machinery

In the manufacturing and construction industries, the employers through their OSH professionals can promote COVID-19 awareness such as the method of spreading, protection, and prevention from the infection. The information can be conveyed via brief meetings, posters and intranet. OSH professionals can also observe the compliance of social distancing, personal hygiene, and PPEs among the workers. Moreover, the OSH professionals must identify the high-risk workers that can get severe complications from the COVID-19 infection. High-risk workers are recommended to work from home. The common contact surfaces such as table, chair, doorknob, handrail, lift button, handle and hand tool, need to be cleaned regularly. Based on the hierarchy of controls, engineering control is more effective than the administrative control and PPE control. The engineering control includes; installing high-efficiency air filters, increasing ventilation rates in the workplace and installing physical barriers. Furthermore, high humidity and high temperature can reduce the transmission rate of COVID-19 [40]. Hence, the humidity level of 40%–70% must be implemented in the workplace.

In the manufacturing industry, employers and OSH professionals must ensure the safety of the workers by implementing COVID-19 control measures. Upon arrival at the workplace, the body temperature of the workers must be checked at the entrance facility. The number of access points can be increased to reduce congestion during the temperature check. Then, each worker is required to clean their hands with hand sanitiser upon leaving the entrance facility. The attendance system that requires physical contact must be removed. According to Refs. [38], an elevated concentration of COVID-19 is measured in the confined space. Hence, adequate ventilation must be provided in confined spaces and rooms, at least 4 times per day. Moreover, the workers must be provided with PPEs such as the face masks and hand gloves, correspond to the OSH regulation. Meanwhile, at the working site, social distancing must be observed and physical contact must be reduced. Crowded canteen during breaks and lunch hours is not allowed. Each worker should sit 1 m apart while eating. If not possible, acrylic panels can be installed on the tables to separate the workers. Furthermore, a shift start time can be delayed for the cleaning and disinfection of equipment. If not possible, equipment sharing must be reduced to a minimum. The shift swapping is not allowed to reduce COVID-19 transmission. Furthermore, the foreign workers must be screened as the infection within them can contribute to a major outbreak [41].

The construction industry is one of the largest and dangerous industries in the world, especially in the United States (US) [42]. Because of that, more control measures should be taken at the construction sites to prevent the spreading of COVID-19. The safety and health officers (SHO) and safety site supervisors (SSS) are responsible to ensure the workers adhere to OSH regulations and COVID-19 procedures. Posters and signages must be installed at appropriate places to promote social distancing and personal hygiene. Moreover, the geofencing system can be used to monitor the number of workers and visitors on the construction sites. This will allow the employer to control and manage the large crowd in the construction site. The temperature gun or scanner can be installed at each site entrance to measure the body temperature of the workers. Workers with COVID-19 symptoms will be denied entry and must seek medical attention immediately. The number of site entrances could be increased to reduce congestion during the temperature check. The start and finish times can be altered to reduce interactions among the workers. The toolbox meeting must be short, to reduce close conversation among them. At the working site, the workers must adhere to social distancing and avoid physical contact. The skin-to-skin works should not be carried out during the outbreak [43]. If not possible, the exposure time between each work must be minimised. Moreover, ventilation must be increased in confined spaces. All windows in the room must be opened to allow good air circulation. The common contact surfaces such as tables, chairs, doorknobs, handrails, lift buttons, hoist buttons, handles and hand tools, must be sanitised regularly. During the lunch break, each worker should sit 1 m apart while eating. Only wrapped and pre-packed foods are allowed in the canteen. The workers are recommended to bring the pre-prepared meal and refillable drinking bottle from home. A special tap mechanism for water dispensers must be designed to ensure its cleanliness [44]. The dining tables must be cleaned between each use. Furthermore, toilet facilities must be sanitised and cleaned regularly. The number of workers in the toilet facility at a time must be restricted. Moreover, the hand washing facilities must be installed at appropriate places with extra supplies of soap, hand sanitiser and paper towels. Sufficient rubbish bins must be provided for wastes disposal. Bathroom facility should be provided to allow workers to clean themselves before going home. Furthermore, the COVID-19 preparedness and response team (CPRT) must be formed to prepare an emergency response plan (ERP) if the worker is suspected of COVID-19 infection. If a worker shows symptoms in the construction site, prompt action must be taken by isolating them in a dedicated room. This dedicated room must have good ventilation and necessities for the suspected worker. After that, the employer must contact the local health authority to transfer the suspected worker from the construction site to the hospital. Then, the construction site will be closed for disinfection. All workers are required to be diagnosed to trace the COVID-19 infection. The construction site can resume operation after being cleared by the health authority.

Machines and equipment must be sanitised and cleaned regularly. Equipment sharing is not recommended to minimize COVID-19 transmission. The drivers of loading vehicles are recommended to remain in the vehicles, and must clean their hands when loading and unloading goods. Contactless communication such as walkie talkie and mobile phones are preferred instead of a close conversation. The electronic paperwork must replace the traditional paperwork in order to reduce physical contact between workers. Furthermore, the workers must be divided into small groups and group members are not interchangeable. The workers must be cross-trained and alternative sources must be identified to facilitate works that require specific skills.

### 3.4 Health care services

The health care workers must clean their hands with water and soap before putting the face mask, face shield and gown on or when taking them off. The reusable PPEs must be cleaned after each use and cannot be shared with other workers. Meanwhile, the single use PPEs must be disposed of after each use in the biomedical waste bin.

The health care service providers such as the hospitals and clinics must limit the number of health care workers engaged with the patients [45]. Moreover, the number of patients in health care facilities must be minimised to reduce COVID-19 transmission. Only patients with appointments are allowed in the facilities. Furthermore, the patients must be punctual to their appointment time. The patients must go home immediately after consultation with their physician.

The health care service providers should provide a dedicated place to evaluate persons with COVID-19 symptoms. This can reduce the close contacts between the non-COVID-19 patients, suspected COVID-19 patients and health care workers. Moreover, persons with COVID-19 symptoms are advised not to visit hospitals or clinics without prior notice. Furthermore, COVID-19 patients must be treated separately from other patients. The dedicated wards for COVID-19 patients must comply with the WHO guidelines. Sufficient ventilation must be provided to each COVID-19 patient (288 m^3^/h) to avoid airborne transmission [46].

If the health care facilities are overcrowded with COVID-19 patients, then the geofencing system can be used to ensure the COVID-19 patients remain at home for self-isolation and reduce the risk of the community from getting infected. For the high-risk group, stocking up medication is recommended to avoid frequent outings. Other than that, hospitals, clinics and pharmacies can deliver medications to their home.

Temporary accommodation should be provided to the health care workers as they are not recommended to go home, to avoid transmitting COVID-19 to their family. The health authority must ensure the welfare of health care workers is not overlooked. Hence, the health authority should provide food and essentials to them.

### 3.5 Elderly care

The older person is included in the high-risk group recommended by the WHO. Moreover, the older person may have the pre-existing conditions which can cause the severe implications if infected with COVID-19. In Sweden, 90% of the death cases were reported from the patients over 70 years of age. Half of them came from the care homes, while another quarter came from their own homes [47]. Because of that, the older person must be protected from contacting the COVID-19 infection.

The temperature gun or scanner must be used to measure the body temperature of the care workers, older persons and visitors at the entrance. Upon arrival at the entrance, the entire body, clothes, shoes and belongings will be sprayed with sanitiser for disinfection. The care workers, older persons and visitors with COVID-19 symptoms will be denied entry and must seek medical attention immediately. The number of visitors will be limited in order to reduce interaction with the outside community. All activities and events must be conducted in small groups to reduce close contact and close conversation. The mealtimes must be rescheduled to avoid mixing between them. Moreover, the disposable plates, cups and cutlery are preferred to reduce object sharing between the residents. The physical contacts and activities between the older persons and care workers must be reduced. The care workers must wear an adequate PPE such as the apron, face shield and face mask in the care home. Meanwhile, the older persons must wear a face mask. The older persons are recommended to stay in the care home to reduce exposure to the outside community. If not possible, the people who live with the older persons must minimize the physical and close contacts with the outside community. Moreover, the medical services can be provided in the care home instead of at the hospital, while medication must be taken at the right time.

### 3.6 Bodies management

The funeral must be conducted and attended by the close relatives. Only a limited number of visitors is allowed to attend the funeral. The social distancing and personal hygiene must be observed during the funeral. Moreover, the bodies must be certified free from COVID-19. The bodies of normal death cases must be screened for COVID-19 infection by the medical practitioner and only being given to the family if free from the disease. If the deceased is tested positive of COVID-19, then the body will be managed by the authority. This is to ensure no COVID-19 related deaths are undetected and to protect those who are managing the bodies. These persons can be infected from the deceased since there is an evidence that the virus can appear on the surface of the bodies [48].

## 4 Conclusion

A war against COVID-19 is far from over. Therefore, it is important for us to prepare for the next waves of COVID-19 infections. The movement restriction is an effort to flatten the infection curve, but not to stop the virus entirely. The social and economic sectors must be opened in phases to reduce the impact of economic recession. Hence, this paper proposes and recommends the new normals and procedures for us to resume our daily activities and works. The community must adapt and embrace the new normal, self-regulation and self-discipline to prevent the spreading of COVID-19. Therefore, the social distancing and personal hygiene measures will remain in place until an effective vaccine is found.

## Ethical approval

No ethical approval required.

## Sources of funding

This research did not receive any specific grant from funding agencies in the public, commercial, or not-for-profit sectors.

## Author contribution

Shahrizan Jamaludin, Nor Azali Azmir, Ahmad Faisal Mohamad Ayob, Nasharuddin Zainal: Writing original draft, Resources. Shahrizan Jamaludin: Editing drafts.

## Guarantor

Nor Azali Azmir – azali@uthm.edu.my.

## Data statement

The data in this paper is not sensitive and not confidential in nature and is accessible in the public domain.

## Provenance and peer review

Not commissioned, externally peer reviewed.

## Consent

No consent required.

## Declaration of competing interest

No conflict of interest.
